# Fifty years of fat: news coverage of trends that predate obesity prevalence

**DOI:** 10.1186/s12889-015-1981-1

**Published:** 2015-07-10

**Authors:** Brennan Davis, Brian Wansink

**Affiliations:** Marketing at the Orfalea College of Business at Cal Poly San Luis Obispo, Grand Ave, 93407 San Luis Obispo, CA USA; Marketing in the Department of Applied Economics, Management at Cornell University, 114 Warren Hall, 14853 Ithaca, NY USA

**Keywords:** Obesity, Mass media, Public health, Marketing analytics, Epidemiology

## Abstract

**Background:**

Obesity prevalence has risen in fifty years. While people generally expect media mentions of health risks like obesity prevalence to follow health risk trends, food consumption trends may precede obesity prevalence trends. Therefore, this research investigates whether media mentions of food predate obesity prevalence.

**Methods:**

Fifty years of non-advertising articles in the *New York Times* (and 17 years for the *London Times*) are coded for the mention of less healthy (5 salty and 5 sweet snacks) and healthy (5 fruits and 5 vegetables) food items by year and then associated with annual obesity prevalence in subsequent years. Time-series generalized linear models test whether food-related mentions predate or postdate obesity prevalence in each country.

**Results:**

United States obesity prevalence is positively associated with *New York Times* mentions of sweet snacks (b = 55.2, CI = 42.4 to 68.1, *p* = .000) and negatively associated with mentions of fruits (b = −71.28, CI −91.5 to −51.1, *p* = .000) and vegetables (b = −13.6, CI = −17.5 to −9.6, *p* = .000). Similar results are found for the United Kingdom and *The London Times*. Importantly, the “obesity followed mentions” models are stronger than the “obesity preceded mentions” models.

**Conclusions:**

It may be possible to estimate a nation’s future obesity prevalence (e.g., three years from now) based on how frequently national media mention sweet snacks (positively related) and vegetables or fruits (negatively related) today. This may provide public health officials and epidemiologists with new tools to more quickly assess the effectiveness of current obesity interventions based on what is mentioned in the media today.

## Background

In both the United States (US) and the United Kingdom (UK), the rate of obesity among adults has risen, from 13.4 % in 1960 to 33.8 % in 2010 in the US and from 15 % in 1993 to 25.4 % in 2010 in the UK [1, 2]. Over a 50-year period (1960–2010), this research investigates whether media mentions of different foods precede or follow obesity prevalence for the US and the UK. The health literature expects the media to follow health risk trends [3]. However, since societal food consumption trends have been shown to predate obesity prevalence increases [4, 5], it may be possible that media mentions of those trends predate actual obesity trends. If food-related mentions in newspapers predate obesity prevalence, tracking key words could help public health officials anticipate future obesity levels (e.g., in response to a range of complex national-level interventions [6]) which might otherwise take years to assess with obesity prevalence measures [7, 8], especially given criticism of many interventions’ efficacy [9].

Newspapers and other media provide time-stamped snapshots of cultural trends [10], including those associated with food and health. By analyzing their coverage of the trends for 50 years, there is an opportunity to observe trends that may be associated with obesity. For example, if the culture has become increasingly obsessed with sugary foods – and less enthusiastic about vegetables during the same time – these would be reflected in the number of news stories mentioning them. If the trends in obesity prevalence follow cultural food trends, then there would be a positive association between news stories of sugary foods and higher obesity prevalence and a negative association between news stories of vegetables and higher obesity prevalence. Therefore, fifty years of newspaper articles would be a valuable public record of cultural trends that predate the obesity rise. Because many social and cultural trends can quickly change, mentions of foods and how they fit in current trends could foreshadow the movement in obesity trends in upcoming years.

Finally, it may be helpful to investigate media mention patterns of obesity comorbidities, especially if the patterns are in contrast with what the medical community thinks to be most critical. Any such contrast, error, or misrepresentation could valuably direct the medical community or public health institutions to alter their emphasis on the various comorbidities associated with obesity prevalence.

This study examines whether media mentions of common foods – sweet snacks, salty snacks, fruits, or vegetables – are associated with national obesity prevalence. It then tests whether these media mentions predate or follow obesity prevalence. Finally, it examines patterns of media mentions of obesity comorbidities.

## Methods

To investigate media mentions in this observational study, annual counts of articles, excluding advertising, were examined from the archives of both the *New York Times* and the *London Times*. Changes in article mentions were compared to obesity prevalence patterns in the US from 1960 (the first year both US obesity prevalence and *New York Times* media mentions were available) to 2010 (i.e., fifty years) and in the UK from 1993 (the first year both UK obesity prevalence and *London Times* media mentions were available) to the same year, 2010. Although not fully representative of their countries, the newspapers considered were nevertheless influential and were also the only ones where all issues were fully-indexed online and available over a significant time period. The *New York Times* had a wide daily circulation (950,000 copies) and won more Pulitzer Prizes than any other newspaper [11]. Including pass-along readership and on-line readership, over 57 % of men and 52 % of women in the US read a daily newspaper [12].

One objective way to assess media coverage is to examine the number of times a particular word or term – like “lettuce” or “potato chips” – was mentioned in the average newspaper story. While this does not capture the manner or valence in which it used, it is an objective measure of usage and exposure. Furthermore, because the total number of articles can vary year-to-year, using the percentage of articles mentioning a term enables a fair comparison to be made across years.

A research assistant identified each term’s media mention by searching the online archives of non-advertising articles provided by the newspapers on their websites. All non-advertising articles mentioning the term counted, regardless of context. The research assistant’s search was validated by calling the *New York Times* and e-mailing the *London Times* to ask for counts of a sample of terms (they did not have time to complete the task for all terms) to compare with the counts retrieved using the online search tools provided on these newspaper’s websites. In all cases, they matched. The assistant used quotation marks if two words were separated by a space (e.g., “potato chips”) within the date range of January 1 to December 31 for each of 50 years from 1960 to 2010. This time scope was used because online data from the newspapers were available for this time period. The plural term was used for fruits and vegetables in order to increase the likelihood of an article’s use of the term as food. For example, an article with “oranges” was more likely to be an article about the fruit versus one with “orange,” which may be referring to the color. Terms were searched without considering compounds so that words like “corn” did not tally counts of “popcorn” or vice versa.

Fruit and vegetable mentions were examined as two primary categories of healthy foods, and sweet and salty snack mentions as two primary categories of unhealthy food [13, 14]. To determine the four categories that represent food consumption extremes – fruits, vegetables, sweet snacks, and salty snacks [13] – the researchers relied on reports from government agencies to determine which foods belong to each category. For example, the United States Department of Agriculture’s (USDA) most recent report on agricultural consumption [14] listed the top five fruits consumed in the US—oranges, apples, grapes, bananas, and pineapples—and the top five vegetables—lettuce, corn, onions, carrots, and cucumbers. The United States Department of Commerce (USDC) listed the top five sweet snack food transactions in the US—cookies, chocolate, candy, cake, and ice cream—and the top five salty snack foods—potato chips, tortilla chips, crackers, popcorn, and pretzels [15]. Since items like pretzels and crackers could be perceived as either sweet or salty, analyses were conducted with and without each item; the results did not differ.

A generalized linear model with a logit link was used to determine the association between annual obesity prevalence, as the dependent variable, and the percentage of articles mentioning vegetables, fruits, salty snacks and sweet snacks, as the independent variables. Actual obesity prevalence in the United States came from waves of the National Health and Nutrition Examination Survey published by the National Center for Health Statistics for select years from 1960 to 2010 [16].

The researchers investigated whether media mentions predated or followed obesity prevalence with three-year lagged time-series analyses [17]. Thus, these were analyses just as before, but with three-year lags for each of the independent variables in the model. Obesity prevalence was associated with the percentage of articles mentioning vegetables, fruits, salty snacks, and sweet snacks three years prior, thus testing whether media mentions followed obesity changes. The reverse was also run; that is, the number of articles mentioning each of the food categories was associated with obesity prevalence three years in the past, thus testing whether obesity changes preceded media mentions. Last, all of these models were compared simultaneously using seemingly unrelated regression (SUR) analyses to see which models had the highest R-Square estimates, investigating which models might best explain the relationships: those where changes in obesity follow changes in media mentions, or those where changes in obesity precede changes in media mentions. Models compared in the SUR analyses included the same number of independent variables.

To determine whether this phenomenon was unique to the US, an informative point of comparison would be a change over time relative to that of another English-speaking country, the UK. To accomplish this, similar media mention analyses were conducted for the *London Times* from 1993 to 2010 using British-equivalent terms (i.e., chips = crisps; candy = sweets; cookies = biscuits, etc.). The time period of 1993 to 2010 was selected because obesity and media mention data prior to this time period were not fully available.

Similar GLM analyses were employed using *London Times* article mentions and obesity outcomes in the UK, using the top snack items because they differed from the US in name only while top fruits and vegetables differed in actual foods consumed. Analyses include UK obesity prevalence since 1993 – the earliest official records reported on obesity prevalence in the UK.

Actual obesity prevalence in the UK came from the National Health Service Health Survey, Department of Health (2011) [18]. Because of the reduced range of obesity data for the UK, the US data were presented first.

Finally, media mention patterns of the word “obesity” and other comorbidity terms were reported. The researchers included mentions of diabetes, high blood pressure, high cholesterol, and hypertension because these comorbidities were highly associated with obesity [2].

## Results

**Fig. 1:**
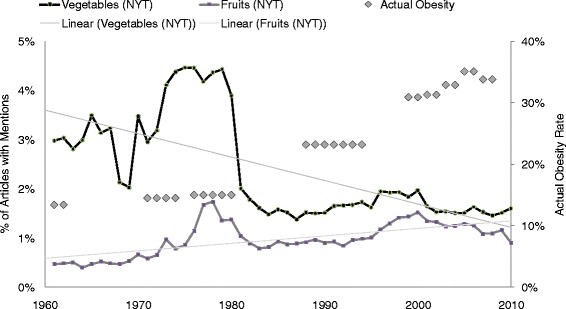
Number of *New York times* articles mentioning fruits or vegetables

**Fig. 2:**
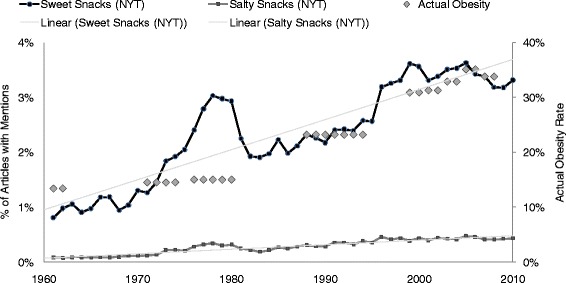
Number of *New York Times* articles mentioning sweet or salty snacks

Articles mentioning vegetables declined by 46 %, and articles mentioning fruits, salty snacks, and sweet snacks increased (92 %, 417 %, and 310 %) over the last 50 years in the *New York Times* (Figs. 1 and 2). Actual US obesity prevalence rose from 13.4 % to 33.8 % over fifty years. Tables 1 and 2 show article US and UK counts respectively (in addition to their percentage of the number of total articles) for the individual sweet and salty snack terms reported at ten-year snapshots over five decades, in order to illustrate simple, descriptive trends.

**Table 1:** Counts (Percentages) of *New York Times* Articles Mentioning the Most Common Snacks

	1960	1970	1980	1990	2000	2010	Percent change 1960 to 2010
Sweet snacks							
Cookies	84 (.06)	113 (.11)	167 (.21)	208 (.22)	356 (.38)	481 (.42)	+473 % (+630 %)
Chocolate	179 (.12)	185 (.18)	548 (.67)	530 (.55)	905 (.97)	1,183 (1.04)	+561 % (+743 %)
Candy	395 (.27)	519 (.50)	612 (.75)	413 (.43)	646 (.70)	916 (.80)	+132 % (+196 %)
Ice cream	232 (.16)	185 (.18)	394 (.48)	426 (.44)	653 (.70)	746 (.65)	+222 % (+310 %)
Cake	340 (.23)	342 (.33)	665 (.82)	514 (.53)	751 (.81)	880 (.77)	+159 % (+230 %
Salty snacks							
Potato chips	17 (.01)	17 (.02)	45 (.06)	78 (.08)	68 (.07)	83 (.07)	+388 % (+523 %)
Tortilla chips	0 (.00)	0 (.00)	4 (.00)	14 (.01)	21 (.02)	34 (.03)	N/A
Crackers	41 (.03)	34 (.03)	104 (.13)	66 (.07)	128 (.14)	118 (.10)	+188 % (+267 %)
Popcorn	36 (.02)	51 (.05)	87 (.11)	104 (.11)	160 (.17)	254 (.22)	+606 % (+800 %)
Pretzels	10 (.01)	11 (.01)	17 (.02)	10 (.01)	29 (.03)	55 (.05)	+450 % (+602 %)
Total # of articles	145,547	102,884	81,303	96,190	92,909	114,110	

Note: Numbers in parentheses are percentages of the total articles that contain the individual terms

**Table 2:** Counts (Percentages) of *London Times* Articles Mentioning the Most Common Snacks

	1960	1970	1980	1990	2000	2010	Percent change 1960 to 2010
Sweet snacks							
Biscuits	198 (.25)	163 (.19)	113 (.15)	187 (.22)	305 (.33)	736 (.72)	+272 % (+188 %)
Chocolate	192 (.24)	205 (.24)	161 (.22)	365 (.44)	1,035 (1.11)	2,343 (2.29)	+1,120 % (+845 %)
Sweets	77 (.10)	76 (.09)	48 (.06)	144 (.17)	284 (.31)	466 (.45)	+505 % (+369 %)
Ice cream	98 (.12)	78 (.09)	81 (.11)	196 (.23)	508 (.55)	946 (.92)	+865 % (+647 %)
Cake	502 (.63)	342 (.40)	208 (.28)	330 (.39)	727 (.78)	1,583 (1.54)	+215 % (+144 %)
Salty snacks							
Potato crisps	79 (.10)	28 (.03)	11 (.01)	50 (.06)	189 (.20)	413 (.40)	+423 % (+305 %)
Crackers	28 (.04)	21 (.02)	23 (.03)	59 (.07)	115 (.12)	171 (.17)	+511 % (+373 %)
Tortilla crisps	0 (.00)	0 (.00)	0 (.00)	0 (.00)	0 (.00)	0 (.00)	N/A^a^
Popcorn	1 (.00)	1 (.00)	9 (.01)	27 (.03)	83 (.09)	272 (.27)	N/A
Pretzels	0 (.00)	0 (.00)	1 (.00)	0 (.00)	8 (.01)	22 (.02)	N/A
Total # of articles	79,369	86,264	74,121	83,691	92,909	102,523	

^a^ Results are similar for all years’ search of “tortilla chips” in the ‘London Times

Note: Numbers in parentheses are percentages of the total articles that contain the individual terms

Otherwise, results reported mentions collected annually as percentages of articles.

### Media mentions and obesity prevalence

Obesity prevalence was significantly associated with lower percentage of articles mentioning vegetables (b = −13.6, 95 % confidence interval [CI] = −17.5 to −9.6, *p* = .000) and fruits (b = −71.28, CI −91.5 to −51.1, *p* = .000). It was also significantly associated with a higher percentage of articles mentioning sweet snacks (b = 55.2, CI = 42.4 to 68.1, *p* = .000). Obesity prevalence was not significantly associated with the percentage of articles mentioning salty snacks between 1960 and 2010 (b = 53.94, CI = −51.1 to 159.0, *p* = .310).

Results of the first seemingly unrelated regression (SUR) analysis revealed that a model in which obesity followed media mentions of vegetables, fruits, sweet snacks and salty snacks three years later (henceforth, “obesity followed mentions”; R^2^ = .94, Chi^2^ = 795.4, *p* = .000) explained more variance than one in which obesity preceded mentions (R^2^ = .88, Chi^2^ = 328.81, *p* = .000). Specific to each, a model in which obesity followed mentions of sweet snacks (R^2^ = .71, Chi^2^ = 145.2, *p* = .000) explained more than a model in which obesity preceded mentions of sweet snacks (R^2^ = .63, Chi^2^ = 122.0, *p* = .000). Likewise, models in which obesity followed mentions of salty snacks (R^2^ = .78, Chi^2^ = 199.9, *p* = .000), vegetables (R^2^ = .43, Chi^2^ = 42.5, *p* = .000), and fruits (R^2^ = .37, Chi^2^ = 36.8, *p* = .000) explained more than counterpart models in which obesity preceded mentions (salty snacks: R^2^ = .70, Chi^2^ = 157.3, *p* = .000; vegetables: R^2^ = .40, Chi^2^ = 47.4; fruits: R^2^ = .21, Chi^2^ = 27.9, *p* = .000). Results were similar for two-, four-and five-year lags (data not shown).

### A global comparison of media mentions

The percentage of *London Times* articles mentioning sweet snacks (b = 42.0, CI = 17.5 to 66.5, *p* = .000) and the percentages of those mentioning salty snacks (b = 175.9, CI = 60.8 to 291.0, *p* = .000) were significantly associated with higher UK obesity prevalence. Likewise, models where obesity followed media mentions (R^2^ = .85, Chi^2^ = 77.0) explained more variance than models where obesity preceded media mentions (R^2^ = .39, Chi^2^ = 8.7). Overall, when compared to the *New York Times*, the results were similar to the trends reported over the past 50 years as well as a restricted analysis of US data from 1993 to 2010.

### Media mentions of obesity and comorbidities

**Fig. 3:**
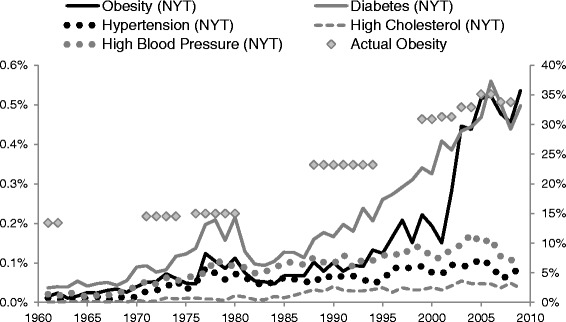
Number of *New York Times* articles mentioning obesity and related comorbidities

Figure 3 shows obesity prevalence and its relation to the mention of “obesity” and comorbidities for the *New York Times*; patterns were similar for the *London Times*. Diabetes was mentioned more than any other comorbidity in both the *New York Times* (0.49 % of articles) and the *London Times* (0.21 % of articles), and over the past 50 years its mention increased 0.43 % in the *New York Times* (*p* = .00) and 0.26 % in the *London Times* (*p* = .00). High blood pressure, high cholesterol and hypertension were mentioned less overall and had the smallest increases over time for both newspapers. Overall, the *New York Times* increased its collective mentions of comorbidities (0.71 % vs. 0.41 %) almost twice the rate of the *London Times* since 1960 (*p* = .00).

## Discussion

This study investigates whether media mentions of food accurately reflect trends related to obesity prevalence. While multiple individual, family, and cultural inputs relate to the complex problem of obesity [4], this research shows that media mentions of unhealthy food are positively associated with obesity prevalence, and media mentions of healthy food are negatively associated with obesity prevalence. It also demonstrates that obesity prevalence increases may follow trends that newspapers report on foods such as sweet snacks, salty snacks, fruits, and vegetables.

This study has limitations worth discussing. Foremost, it makes no claims of causality as an inherent limitation of analyzing secondary data. However, it presents models where obesity prevalence follows versus precedes media trends to see which explains more variance. The models are neither exhaustive nor causal. Some food terms used in searches may have minor non-food contexts. For example, popcorn might be used in an article describing Styrofoam packing materials for shipping. Also, the valence of the terms’ uses is unknown, though this study presents an objective way of measuring exposure. Data were collected only from two major newspapers, so these findings cannot be overly generalized to other countries or regions within the US or UK where people do not read the particular newspapers studied here.

This study could be valuable to the medical and public health community in helping them analyze and adjust public health messages and interventions. The results demonstrate trends in the culture that predate obesity prevalence over a 50-year period of time. Since few research measures exist from the earlier decades when obesity prevalence grew most rapidly, this analysis of newspaper articles over 50 years may provide valuable insights to help combat obesity in the future.

## Conclusion

It may be possible to estimate a nation's future obesity prevalence based on how frequently national media mention sweet snacks (positively related) and fruits or vegetables (negatively related) today. This provides public health officials and epidemiologists with new tools to more quickly assess the effectiveness of current obesity interventions. In doing so, it could help them to more accurately analyze and adjust public health messages and interventions. If we wish to estimate obesity rates three years from now, an indicator may be what is mentioned in newspapers today.
